# Neoadjuvant, Perioperative, and Adjuvant Immunotherapy in Early-Stage Surgically Resectable Non-Small Cell Lung Cancer: Updates and Future Perspectives

**DOI:** 10.3390/cancers17132077

**Published:** 2025-06-21

**Authors:** Diamone Gathers, Cameron Oswalt, Laura Alder, Kevin Chen, Jordyn P. Higgins, Jeffrey M. Clarke, Eziafa I. Oduah

**Affiliations:** 1 Duke University Health Systems, School of Medicine, Duke University, Durham, NC 27708, USAcameron.oswalt@duke.edu (C.O.); laura.alder@duke.edu (L.A.);; 2 Duke Cancer Institute, Durham, NC 27710, USA; 3 Lineberger Comprehensive Cancer Center, University of North Carolina, Chapel Hill, NC 27514, USA; kevin.chen2@unchealth.unc.edu

**Keywords:** non-small cell lung cancer (NSCLC), immunotherapy, early-stage NSCLC, neoadjuvant, perioperative, adjuvant

## Abstract

Historically, systemic therapy for resectable non-small cell lung cancer (NSCLC) has been associated with a modest impact on overall survival. The current treatment options for early-stage resectable NSCLC include neoadjuvant, adjuvant, and perioperative immunotherapy in combination with chemotherapy. In this review, we explore the current treatment paradigms and emerging opportunities for improved survival outcomes from using immunotherapeutic approaches in the treatment of early-stage resectable NSCLC. The incorporation of immunotherapy into neoadjuvant, adjuvant, and perioperative treatment of surgically resectable NSCLC has yielded improved outcomes beyond chemotherapy-alone approaches. Despite this, there remains a margin for improving survival outcomes for patients. Clinical trials utilizing novel agents and approaches that modulate the anti-tumor immune response are currently ongoing and will likely inform the future treatment landscape for early-stage surgically resectable NSCLC.

## Simple Summary

Immunotherapy has changed the landscape of non-small cell lung cancer (NSCLC) treatment. Although initially studied in the advanced/metastatic setting, recent trials have demonstrated impressive benefits in early-stage disease. This review will focus on the various immune checkpoint inhibitor regimens that have been studied in and approved for surgically resectable NSCLC patients and outline the unanswered questions in the field and highlight the vital role of multi-disciplinary discussions in this patient population.

## 1. Introduction

Lung cancer remains the leading cause of cancer mortality in the United States and worldwide. In 2024, there was an estimated 234,580 new cases with approximately 125,070 deaths [1,2]. Approximately 30% of patients with non-small cell lung cancer (NSCLC) are diagnosed at an early stage; at this stage, curative surgery is the treatment of choice [3,4,5,6]. However, recent outcome data from the Surveillance, Epidemiology, and End Results (SEER) registry indicate that the 5-year survival for patients with localized NSCLC is 60% and is as low as 37% for those with regional nodal metastasis [7]. These statistics demonstrate the significant risk of recurrence and mortality from lung cancer after curative surgery, highlighting the need to incorporate advanced systemic therapies into the treatment paradigm for surgically resectable NSCLC.

The initial efforts to improve survival for surgically resectable NSCLC focused on the use of post-operative (adjuvant) or pre-operative (neoadjuvant) platinum-based chemotherapy regimens. Studies have shown that the use of adjuvant chemotherapy in patients with early-stage surgically resected NSCLC had an overall modest clinical impact on survival [1]. The lung adjuvant cisplatin evaluation (LACE) meta-analysis showed that adjuvant cisplatin improved overall survival (OS) by 5.4%, showing benefit primarily in Stage II–IIIA patients [8]. Although some studies of neoadjuvant chemotherapy were promising compared to the available adjuvant data, statistically significant OS benefits were not demonstrated. Moreover, some trials were aborted early when survival data on the adjuvant approach emerged. With the advent of immunotherapy and targeted therapies in the last decade, there have been strong efforts to incorporate these agents into the treatment landscape for surgically resectable NSCLC to improve survival.

The activation of the anti-tumor immune response through immune checkpoint inhibition was found to be effective in metastatic lung cancer, paving the way for using this approach in the treatment of earlier stages of NSCLC [9,10,11,12,13]. The addition of immunotherapy into neoadjuvant (inductive or pre-operative) systemic therapy for early-stage NSCLC yielded improved outcomes in patients. However, the rate of pathologic complete responses, a surrogate for long-term survival, with the post-surgical regimes remained below 30% [14,15,16]. Adjuvant (postoperative) and perioperative approaches using immunotherapy combinations are now also standard of care for select early-stage NSCLC patients. Despite these significant advances in the treatment of early-stage NSCLC, unanswered questions remain and debates continue over the utility of neoadjuvant versus perioperative or adjuvant only mmunotherapeutic approaches.

In this narrative review, the data from key clinical trials incorporating neoadjuvant, adjuvant, and perioperative immunotherapy strategies that have shaped the current treatment landscape in early-stage NSCLC without actionable EGFR and ALK alterations are described and are summarized in Table 1. Current challenges, ongoing studies, and opportunities for future research that will shape the future of immunotherapy in early-stage NSCLC are also discussed.

## 2. Neoadjuvant Chemotherapy and Immunotherapy Approaches

The evaluation of neoadjuvant systemic therapy for the treatment of early-stage surgically resectable NSCLC predates the era of immunotherapy. Initial studies of neoadjuvant systemic therapies investigated the addition of platinum-doublet chemotherapies prior to surgery. In a Spanish phase III study, 624 patients with Stage IA to Stage IIIA NSCLC were randomized into neoadjuvant chemotherapy followed by surgery, surgery alone, and surgery followed by adjuvant chemotherapy groups. There were no significant differences in 5-year disease-free survival (DFS) between the neoadjuvant and adjuvant chemotherapy arms. The 5-year DFS rate was 38.3% in the preoperative population vs. 34.1% in the postoperative population with a hazard ratio [HR] for progression or death of 0.92 (*p* = 0.176). The 5-year DFS rate in the adjuvant arm was 36.6% compared to 34.1% in the surgery arm (HR 0.96; *p* = 0.74). It was also noted that a higher proportion of patients in the preoperative arm (90.4%) completed three planned chemotherapy cycles compared to the postoperative arm (60.9%) [29].

Two additional phase III studies were performed to further assess the benefits of neoadjuvant chemotherapy. The CHEST and SWOG 9900 trials both compared neoadjuvant chemotherapy regimens followed by surgery versus surgery alone in patients with Stage IB to Stage IIIA NSCLC [30,31]. The reported results from both studies showed modest efficacy; however, both studies were terminated prematurely due to the emergence of data supporting a greater survival benefit with adjuvant chemotherapy. A meta-analysis of 13 randomized control trials was performed (data ranging from 1985 to 2005 and for Stages IB–IIIA), evaluating the benefit of neoadjuvant chemotherapy versus surgery alone. The overall survival of NSCLC patients in the neoadjuvant chemotherapy arm improved significantly compared with those undergoing surgery alone (combined HR = 0.84; 95% CI: 0.77–0.92; *p* = 0.0001). When only patients with Stage III NSCLC were considered, the result was similar (combined HR = 0.84; 95% confidence interval: 0.75–0.95; *p* = 0.005) [32].

The emergence of immunotherapy onto the therapeutic landscape for metastatic NSCLC paved the way for the consideration of new neoadjuvant treatment approaches. Early data indicated that the use of neoadjuvant immunotherapy in resectable NSCLC was feasible and safe, with no significant compromise in surgical outcomes. In a phase II study of Stage I to Stage IIIA resectable NSCLC, two doses of preoperative nivolumab (3 mg/kg) were administered 2 weeks apart, followed by surgery. Neoadjuvant nivolumab was found to have an acceptable side effect profile and resulted in a major pathologic response (mPR), defined as the presence of no more than 10% viable tumor cells, in 45% of resected tumors [33]. Another earlier study investigated the addition of immunotherapy to chemotherapy in a phase II trial of Stage IB to Stage IIIA resectable NSCLC. Neoadjuvant carboplatin, nab-paclitaxel, and atezolizumab were administered every 3 weeks for two cycles, followed by an interim evaluation. An additional two cycles were given if no progression was detected after the first two cycles, followed by surgery. A total of 30 patients were enrolled (75% with Stage IIIA disease), and 17 of these patients (57%) had an mPR. The treatment was tolerable with manageable side effects, without significant compromise of surgical feasibility [34].

CheckMate (CM) 816 was the first phase III clinical trial to demonstrate the benefits of neoadjuvant immunotherapy in resectable NSCLC. Patients with Stage IB (tumors > 4 cm) to Stage IIIA disease, without EGFR or ALK alterations, were randomized to receive three cycles of neoadjuvant platinum-doublet chemotherapy with nivolumab (360 mg) every 3 weeks, three cycles of platinum-doublet chemotherapy alone every 3 weeks, or dual immunotherapy with three cycles of q3 weekly nivolumab (3 mg/kg) and ipilimumab (1 mg/kg) every 2 weeks. Surgery was performed within 6 weeks of completion of the neoadjuvant therapy. Of note, the dual-immunotherapy arm was closed early after the results of the NADIM and NEOSTAR trials were reported [15,18].

The two primary endpoints of CheckMate 816 were event-free survival (EFS) and pathologic complete response (pCR), defined as 0% residual viable tumor cells in the primary tumor and sampled lymph nodes. In the initial report, the median EFS was 31.6 months (95% confidence interval [CI]: 30.2—not reached [NR]) in the chemotherapy–immunotherapy arm compared to 20.8 months in the chemotherapy-alone arm (95% CI: 14.0–26.7). The pCR rate in the chemotherapy–immunotherapy arm was statistically significantly higher (24.0%; 95% CI: 18.0–31.0) compared to the chemotherapy arm (2.2%; 95% CI: 0.6–5.6) (odds ratio: 13.94; 99% CI: 3.49–55.75; *p* < 0.001). The pCR rates were higher in the patients receiving chemotherapy–nivolumab across all subgroups except in the never smokers. At the first interim analysis, the HR for death in the chemotherapy–immunotherapy arm compared to the chemotherapy-alone arm was 0.57 (99.67% CI: 0.30–1.07), although the median OS had not been reached in either group. When compared to the chemotherapy-alone arm, neoadjuvant chemoimmunotherapy did not negatively impact the ability to perform surgical resection. Approximately 83.2% of the patients in the chemotherapy–nivolumab arm underwent surgery, compared to 75.4% in the chemotherapy-alone arm. Adverse events (AEs) occurred at similar rates in the two groups, with 92.6% of the patients in the chemoimmunotherapy group experiencing at least one AE compared to 97.2% in the chemotherapy-alone group; grade 3–4 treatment-related adverse events occurred in 33.5% of patients receiving immunotherapy compared to 26.9% in the chemotherapy-alone group. In conclusion, CheckMate 816 demonstrated that neoadjuvant chemotherapy plus nivolumab had a significant benefit over chemotherapy alone with respect to EFS and pCR without an increase in adverse events or compromising surgical feasibility [14]. Nivolumab became the first immunotherapeutic to receive Food and Drug Administration approval in combination with platinum-doublet chemotherapy in the neoadjuvant setting for resectable NSCLC in March 2022 [35].

The 4-year survival update of Checkmate 816 demonstrated continued benefit with the addition of nivolumab. The median EFS in the nivolumab arm was 43.8 months compared to the 18.4 months in the chemotherapy-alone arm (HR: 0.66; 95% CI: 0.49–0.90). The 4-year EFS rates were 49% and 38%. There was a 13% improvement in OS in the nivolumab arm compared to the chemotherapy-alone arm at 4 years (71% vs. 58%). The median OS had not yet been reached in either group. Importantly, the patients in the immunotherapy arm who achieved a pCR demonstrated improved OS compared to those who did not. This 4-year analysis demonstrated a survival benefit with the addition of immunotherapy and a sustained EFS benefit over time compared to chemotherapy alone while highlighting the long-term benefits of achieving a pCR [17].

In an exploratory analysis of the dual-immunotherapy arm of CheckMate 816 (three cycles of nivolumab every 2 weeks and one cycle of ipilimumab) compared to chemotherapy, there was improved EFS (54.8 months vs. 20.9 months), a higher 3-year OS rate (73% vs. 61%), and a higher pCR rate (20.4% vs. 4.6%) in the dual-immunotherapy arm compared to the chemotherapy arm. Moreover, there were lower rates of grade 3 or 4 treatment-related adverse events (14% vs. 36%) in the dual-immunotherapy group as well [36]. This data suggests that dual-immunotherapy approaches could be a reasonable approach for patients for whom there is a need to avoid chemotherapy. The dual-neoadjuvant immunotherapy approach was also used in the phase II NeoCOAST trial. The NeoCOAST trial compared durvalumab alone, durvalumab plus oleclumab, durvalumab plus monalizumab, and durvalumab plus danvatirsen followed by surgery. The trial demonstrated that the combination neoadjuvant immunotherapy approaches resulted in higher mPR rates while maintaining similar safety profiles. The enhanced responses were attributed to enhanced immune infiltration, interferon responses, and functional immune cell activation, which were observed through immune profiling analysis [37].

The benefit of neoadjuvant chemoimmunotherapy over neoadjuvant chemotherapy has been clearly demonstrated both in clinical trials and meta-analysis reports. A meta-analysis of neoadjuvant chemoimmunotherapy versus chemotherapy in resectable NSCLC demonstrated improved pooled OS (HR: 0.65), EFS (HR: 0.59), mPR rates (risk ratio: 3.42), and pCR rates (risk ratio: 5.52) for neoadjuvant chemoimmunotherapy over chemotherapy. This analysis also demonstrated that the EFS benefit was sustained even in the PD-L1 < 1% population (HR 0.74) [38]. A similar systematic review and meta-analysis of neoadjuvant chemoimmunotherapy compared to chemotherapy alone demonstrated 2-year EFS and pCR benefits irrespective of PD-L1 status, platinum chemotherapy selection, number of neoadjuvant cycles, or the use of adjuvant immunotherapy. However, their results showed a higher risk of relapse in tumor cells negative for PD-L1 compared to those with low or high PD-L1 scores [39]. As the neoadjuvant treatment landscape continues to evolve, it will be of paramount importance to continue conducting similar analyses to determine the best treatment approaches across different patient and tumor characteristics and the most appropriate trial end points to assess neoadjuvant immunotherapy outcomes.

## 3. Perioperative Checkpoint Blockade in Early-Stage NSCLC

Given the success of including checkpoint inhibitors in neoadjuvant and adjuvant settings (discussed below), multiple studies began to assess a sandwich approach in which immunotherapy was layered post-operatively to patients who received neoadjuvant chemo-immunotherapy strategies.

NADIM was the first trial to test the addition of perioperative checkpoint inhibition in patients with resectable NSCLC [18]. This was a single-arm phase II trial in Spain that enrolled 46 individuals with resectable Stage IIIA NSCLC. Neoadjuvant platinum-doublet chemotherapy combined with nivolumab was given for up to three cycles followed by surgery and adjuvant nivolumab for up to 1 year. Among the 41 patients who underwent surgery, 83.0% (95% CI: 68–93) had a major pathological response, including 63.4% (95% CI: 62–91) who showed a complete pathological response (pCR) [18]. The PD-L1 TPS was significantly higher in patients who had a pCR (*p* = 0.042). Follow-up was concluded at 60 months in July 2023 and showed a 5-year progression-free survival of 65.0% (95% CI: 49.4–76.9) and an overall survival of 69.3% (95% CI: 53.7–80.6) in the intention-to-treat population. Disease progression occurred in 11 patients (24%) [19]. The neoadjuvant treatment did not significantly delay the planned surgery. This initial trial was instrumental in demonstrating the safety and efficacy of perioperative immunotherapy treatment in a population at high risk of recurrence.

In the follow-up to the NADIM trial, the investigators evaluated the perioperative approach in resectable Stage IIIA and IIIB NSCLC. In NADIM II, patients with resectable Stage IIIA/B NSCLC were randomly assigned to two groups in a 2:1 ratio: neoadjuvant platinum-doublet chemotherapy plus nivolumab and SOC neoadjuvant chemotherapy followed by surgery groups. Adjuvant nivolumab was administered to the patients in the experimental arm who achieved R0 resection. Similar to NADIM, the addition of nivolumab increased the pCR rate (37% vs. 7%) (RR: 5.34; 95% CI: 1.34–21.23; *p* = 0.02) [40]

Perioperative pembrolizumab was tested in the KEYNOTE-671 clinical trial. Patients with either Stage II, IIIA, or IIIB disease were assigned to receive neoadjuvant cisplatin-based chemotherapy in combination with pembrolizumab (200 mg) or placebo once every 3 weeks for 4 cycles, followed by surgery and adjuvant pembrolizumab (200 mg) or placebo once every 3 weeks for up to 13 cycles, respectively [41]. At the 24-month follow-up, the EFS rate in the pembrolizumab arm was 62.4% compared to 40.6% in the placebo group (HR for progression, recurrence, or death: 0.58; 95% CI: 0.46–0.72; *p* < 0.001). Like other studies, the inclusion of neoadjuvant pembrolizumab resulted in a higher pCR rate of 18.1% vs. 4.0% in the chemotherapy-alone group (95% CI: 10.1–18.7; *p* < 0.0001). Similarly, 30.2% of the pembrolizumab group achieved an mPR versus 11.0% of the placebo group (95% CI: 13.9–24.7; *p* < 0.0001) [41]. At the second interim analysis, the 36-month OS estimates were 71% (95% CI: 66–76) in the pembrolizumab group and 64% (95% CI: 58–69) in the placebo group (HR: 0.72; 95% CI: 0.56–0.93; *p* = 0.0052). The median event-free survival (EFS) was 47.2 months (95% CI: 32.9–not reached [NR]) in the pembrolizumab group and 18.3 months (95% CI: 14.8–22.1) in the placebo group (HR: 0.59; 95% CI: 0.48–0.72) [20]. Based on these results, pembrolizumab became the first immunotherapy to receive FDA approval for perioperative use in early-stage resectable NSCLC.

CheckMate 77T was another phase III study investigating peri-operative immune checkpoint inhibition [21]. Patients with Stage IIA to IIIB NSCLC received either neoadjuvant nivolumab plus platinum-doublet based chemotherapy or neoadjuvant chemotherapy plus placebo every 3 weeks for four cycles, followed by surgery and adjuvant nivolumab or placebo every 4 weeks for 1 year. At a median follow-up of 25.4 months, the 18-month EFS was 70.2% in the nivolumab group and 50.0% in the chemotherapy group (HR for disease progression or recurrence, abandoned surgery, or death: 0.58; 97.36% CI: 0.42–0.81; *p* < 0.001) [14]. A higher percentage of patients in the nivolumab group achieved a pCR (25.3%) compared to the chemotherapy group (4.7%) (odds ratio: 6.64; 95% CI: 3.40–12.97), similar to the results seen in CheckMate 816 [14]. Both CheckMate 77T and 816 showed that patients who achieved a pCR had significantly better outcomes with improved EFS. A higher percentage of patients also reached an mPR with nivolumab (35.4% vs. 12.1%) (odds ratio: 4.01; 95% CI: 2.48–6.49) [21]. In the landmark analysis of the EFS for each adjuvant treatment using the date of definitive surgery as the landmark timepoint, there was an observed EFS benefit in the patients receiving nivolumab vs. the placebo. Although this was used to suggest the added benefit of nivolumab in the adjuvant phase of treatment, it remains unclear what the contribution of adding adjuvant nivolumab is over using neoadjuvant nivolumab alone, since the comparison was with patients who received neoadjuvant chemotherapy alone. This remains an ongoing question and a debated topic since no trial has reported data to compare the benefit of perioperative immunotherapy over the inclusion of neoadjuvant immunotherapy with chemotherapy alone.

Lastly, the AEGEAN trial assessed the addition of durvalumab to a standard neoadjuvant chemotherapy regimen in patients with resectable NSCLC (Stage II to IIIB [N2 node stage]). The patients were treated with either platinum-based chemotherapy plus durvalumab or placebo, which was administered intravenously every 3 weeks for 4 cycles before surgery, followed by adjuvant durvalumab or placebo intravenously every 4 weeks for 12 cycles [42]. pCRs occurred at a higher rate in the durvalumab arm (17.2%) vs. the placebo arm (4.3%) (95% CI: 8.7 to 17.6; *p* < 0.001). The EFS at 12 months was also significantly higher in the durvalumab group at 73.4% vs. 64.5% in the placebo group. The EFS and pCR benefits were observed regardless of stage or PD-L1 expression [39]. At the second planned interim analysis, the median EFS was not reached (95% CI: 42.3–NR) with the durvalumab regimen (*n* = 366) compared with 30.0 months (95% CI: 20.6–NR) with the placebo regimen (HR: 0.69; 95% CI: 0.55–0.88) at a median follow-up of 25.9 months (range, 0.0–58.6) in the censored patients. The 3-year EFS rates were 60.1% and 47.9%, respectively [22].

Other studies have also demonstrated the benefit of adding perioperative PD-1 blockade to standard chemotherapy. The phase III NEOTORCH trial randomly assigned resectable Stages II–IIIB (AJCC 8th edition) patients in a 1:1 ratio to receive toripalimab or placebo in combination with platinum-based chemotherapy for three cycles followed by surgery and an additional cycle of adjuvant [23]. Patients then continued toripalimab or placebo maintenance for 13 additional cycles to complete one year of total treatment. The interim analysis for the Stage III disease patients demonstrated a significant benefit with the addition of toripalimab. Major pathologic responses were achieved in 48.5% of the patients in the toripalimab arm versus 8.4% in the placebo arm while the pCR rate in the toripalimab treatment arm was 24.8% compared to 1.0% in the placebo arm. Investigator-assessed EFS was higher in the toripalimab arm (median: NE vs. 15.1mo; HR: 0.40; 95% CI: 0.28–0.57). The safety profile was consistent with prior chemoimmunotherapy combination trials, with the majority of toxicities arising from the chemotherapy treatment. The toripalimab arm did have increased rates of transaminitis, hypothyroidism, and pneumonitis, which are consistent with the known toxicities of ICIs.

Similarly, the RATIONALE 315 trial also demonstrated improved outcomes in patients who received perioperative tislelizumab in addition to platinum-based chemotherapy [24]. This study was conducted in a similar population with slight differences in the treatments. The patients were randomized to receive three to four cycles of neoadjuvant chemotherapy with tislelizumab or placebo, followed by eight 6-week cycles of adjuvant tislelizumab or placebo after surgical resection. More patients received three cycles of neoadjuvant treatment (55%) than four cycles (37%). The addition of tislelizumab to chemotherapy resulted in a significant increase in patients who achieved an mPR (56% vs. 15%) or pCR (41% vs. 6%), as well as an improvement in EFS (HR: 0.56; 95% CI: 0.40–0.79). A trend towards improved overall survival was seen with tislelizumab but was not statistically significant. Treatment-related adverse events were similar in both groups, with the tislelizumab group experiencing more immune-related adverse events (40% vs. 18%) such as skin reactions, pneumonitis, hepatitis, and endocrinopathies.

## 4. Adjuvant Immunotherapy in Early-Stage NSCLC

Adjuvant therapy alone following surgery remains an option for patients who did not receive neoadjuvant systemic therapy. The goal of adjuvant therapy is to eliminate micrometastases and to prevent recurrence. For patients with Stage IB or IIA disease and negative margins, adjuvant chemotherapy is only recommended for those with high-risk features such as poorly differentiated tumors, vascular invasion, wedge resection, visceral pleural involvement, and unknown lymph node status. For patients with Stage IIB to Stage IIIB disease and negative margins, adjuvant chemotherapy is a category 1 recommendation according to the NCCN Guidelines [43]. There are ongoing clinical trials exploring chemotherapy with concurrent or subsequent immunotherapy in patients with resectable NSCLC [44]. In addition, the use of immune checkpoint inhibition has been shown to be able to reverse the surgically induced inflammatory response and enhance the anti-tumor activity of T cells [45,46].

Adjuvant immunotherapy has been investigated in the postoperative setting. PEARLS/Keynote-091 investigated the use of pembrolizumab compared to placebo in early-stage resectable NSCLC after curative surgery with or without adjuvant chemotherapy. The primary endpoints were DFS in the overall population and in the PD-L1 TPS ≥ 50% group. Adjuvant pembrolizumab improved DFS in the overall population compared to the placebo. The median DFS was 58.7 months in the pembrolizumab arm vs. 34.9 months in the placebo arm (HR: 0.73; 95% CI: 0.60–0.89), with estimated 18-month DFS rates of 73.8% for the pembrolizumab group and 63.1% for the placebo group [25]. In the PDL1 ≥ 50% population, the median DFS was not reached in both the pembrolizumab (95% CI: 44.3 to not reached) and placebo groups (95% CI: 35.8 to not reached). Interestingly, there was no statistically significant improvement in median DFS in the pembrolizumab group compared to the placebo in the PDL1 >50% patients (HR: 0.82; 95% CI: 0.57–1.18; *p* = 0.14). In the updated analysis of the patients who received chemotherapy at a median follow-up of 51.7 months, the DFS benefit of pembrolizumab over the placebo was maintained in the ITT population. The 4-year DFS rates in the pembrolizumab and control groups were 57.0% (95% CI: 47.9–65.1%) and 49.1% (95% CI: 39.8–57.8%). There were no statistically significant differences in the median DFS in both arms in the PD-L1 TPS ≥ 50% patient population [26].

In the Impower010 trial, patients with Stage IB–IIIA NSCLC who had received adjuvant chemotherapy after surgical resection were randomized to receive atezolizumab or the best supportive care. In patients with PD-L1-positive Stage II–IIIA disease, atezolizumab showed improved DFS compared to the best supportive care at a median follow-up of 32 months (HR: 0.66; 95% CI: 0.50–0.88; *p* = 0.0039). Moreover, atezolizumab showed improved DFS over the placebo in all Stage II–IIIA patients (0.79; 0.64–0.96; *p* = 0.020) regardless of PDL1 status. In the ITT population, the HR for DFS was 0.81 (0.67–0.99; *p* = 0.040). Atezolizumab-related grade 3 and 4 adverse events occurred in 11% of the 495 patients and grade 5 events occurred in 1% of patients [27]. In the updated overall survival analysis, an OS HR of 0.43 (95% CI 0.24–0.78) was reported in favor of atezolizumab over the placebo in PDL1 >50% Stage II–IIIA patients. In the ITT population, the OS data also showed a trend in favor of atezolizumab but it was not statistically significant and remains immature [28].

Interestingly, the BR31 trial, which examined the benefit of adjuvant durvalumab treatment following surgery and optional adjuvant chemotherapy, found no additional benefit regardless of the PD-L1 thresholds [47], which is contrary to the results of the existing adjuvant ICI trials IMPower010 and Keynote091. Although the administration of adjuvant chemotherapy prior to durvalumab was optional, most patients (84%) received adjuvant chemotherapy in this study. Additional ongoing studies of adjuvant immunotherapy approaches including the IMPower030 and ANVIL/ALCHEMIST trials, which are ongoing and will add to the growing body of literature on the use of immune checkpoint inhibitors in the adjuvant setting (described in Table 2).

## 5. Ongoing Studies

Emerging studies incorporating immunotherapy with immune checkpoint inhibitors are ongoing. The previously discussed phase II platform NeoCOAST trial in which patients were randomized to receive neoadjuvant durvalumab alone, durvalumab + oleclumab (anti-CD73), durvalumab + monalizumab (anti-NKG2A), or durvalumab + danvatirsen (anti-STAT3) showed promising results [37]. mPRs were observed in 11.1% of the durvalumab-alone group, 19.0% of the durvalumab + oleclumab group, 30.0% of the durvalumab + monalizumab group, and 31.3% of the durvalumab + danvatirsen group. Building on this study, the NeoCOAST-2 trial randomized patients to receive neoadjuvant platinum-doublet chemotherapy combined with one of four regimens: durvalumab + oleclumab, durvalumab + monalizumab, volrustomig (bi-specific PD-1/CTLA-4), or durvalumab + datopotamab deruxtecan (TROP2-directed antibody–drug conjugate) for four cycles, followed by up to 1 year of the investigated adjuvant agent after surgery [48]. The interim analysis found pCR rates of 20.0%, 26.7%, and 34.1% and MPR rates of 45.0%, 53.3%, and 65.9% for the durvalumab + oleclumab, durvalumab + monalizumab, and durvalumab + datopotamab deruxtecan arms, respectively. Grade 3 or higher treatment-related adverse events were seen in 31.1%, 29.6%, and 18.5%, respectively, of the patients in these groups. These findings are likely to usher in the next generation of combination treatment regimens in the perioperative space. Ongoing trials are investigating novel combinations with additional immune checkpoint inhibitor targets including anti-LAG-3, anti-IgG1, and anti-BTLA monoclonal antibodies. A key difference in the ongoing trials is the addition of novel immunotherapy targets. For example, the IMPower010 data demonstrated improved 3-year DFS with the use of adjuvant atezolizumab, while IMPower030 (NCT03456063) is measuring the DFS of patients given neoadjuvant and adjuvant atezolizumab vs. a placebo. The ANVIL trial (NCT02595944), as part of the national ALCHEMIST initiative), is seeking to evaluate 10-year DFS and OS in patients treated with adjuvant nivolumab vs. a placebo. A comprehensive list of ongoing clinical trials using immunotherapy in resectable NSCLC is provided in Table 2.

## 6. Discussion

The incorporation of immune checkpoint blockade into the treatment paradigm for ALK/EGFR-negative surgically resectable early-stage NSCLC was a tremendous advance in therapy and patient outcomes. The addition of immunotherapy to neoadjuvant chemotherapy not only improved efficacy but it was well tolerated and did not impact surgical outcomes compared to neoadjuvant chemotherapy alone. The ability to attain a pCR is inarguably one of the most important efficacy and survival indicators following neoadjuvant immunotherapy. However, pCR rates remain close to 20% for all established regimens using anti-PD1 and chemotherapy combinations, increasing the impetus for ongoing and future studies to improve survival in early-stage NSCLC patients.

The perioperative approach involving the layering of checkpoint inhibition post-operatively for a year in patients who received neoadjuvant chemo-immunotherapy aims to improve survival. While the OS read outs from KN671 and CM77T show a clear benefit for adding immune checkpoint inhibition to neoadjuvant chemotherapy, the individual benefit of continuing anti-PD1 treatment postoperatively is difficult to discern due to the study designs. It is likely that there are subsets of patients for whom postoperative immunotherapy would be of incremental benefit. However, there is a lack of prospectively designed studies that focused on identifying molecular markers or clinical features that can define specific subsets of patients that are most likely to obtain benefits from the sandwich approach. The ongoing ADOPT-lung and PROSPECT-lung trials may help elucidate an answer by comparing neoadjuvant vs. perioperative durvalumab and perioperative durvalumab vs. investigators’ choice of pembrolizumab, nivolumab, and atezolizumab, respectively [49,50]. These findings will hopefully help ensure that patients are treated with the most appropriate agents for the optimal duration to avoid overtreatment and unnecessary toxicities, as well as potentially help identify the most appropriate backbone systemic therapy regimen to which novel therapies can be added in future clinical trials.

Another important question is the optimal management for the patients who do not achieve a pCR or MPR, and particularly those with residual mediastinal nodal disease. Peri-operative immunotherapy has demonstrated benefits in these groups, as discussed above; however, these patients remain at high risk of recurrence. There has been a longstanding debate on the use of postoperative radiotherapy (PORT) in this context, with trials showing overall mixed results; they suggest a disease-free survival benefit, but with no improvement in overall survival or disease-free survival [51,52,53]. However, there have been major advances in radiation techniques since these studies were conducted, allowing for safer and more precise delivery strategies. Its possible synergy with neo-adjuvant immunotherapy is also unclear. Overall, more data is needed to identify the optimal treatment(s) for this high-risk population.

Strategies incorporating neoadjuvant immunotherapies are frequently preferred over adjuvant-only (or post-operative) regimens due to the higher rates of completion of planned systemic treatments, earlier treatment of micrometastatic disease, and more robust anti-tumor immune responses in the presence of the tumor [54]. Moreover, the ability to attain a pCR correlates with better survival in clinical trials. However, although uncommon, some patients experience disease progression while on neoadjuvant therapy and may no longer be candidates for definitive surgery. This forms the basis of arguments that favor an adjuvant-only approach. More importantly, it raises the question of the appropriate patient selection method for giving neoadjuvant therapy versus upfront surgery to patients with surgically resectable early-stage disease. The exclusion of patients with driver alterations in EGFR and ALK from studies on neoadjuvant chemo-immunotherapy is an established practice, especially considering the results from the ADAURA and ALINA studies that showed a benefit for adjuvant therapy for these patients [55,56]. Clinical trials investigating neoadjuvant and adjuvant strategies in resectable NSCLC with other actionable molecular alterations including KRAS, ROS1, and RET are currently underway and may yield data that aid in the selection of patients for targeted therapy approaches [57] The role of other nonactionable molecular alterations such as *STK 11*, *KEAP1*, and *TP53* mutations and other known markers of poor responses to ICIs in determining the appropriateness of neoadjuvant immunotherapies vs. adjuvant and perioperative approaches is another area for investigation.

The role of neoadjuvant chemoimmunotherapy in borderline resectable patients for downstaging is controversial. The clinical trials for the currently approved regimens only included surgically resectable candidates at the time of enrollment; however, there is data demonstrating the potential for downstaging after perioperative treatments [58]. For example, in the Checkmate 816 study, the patients who received chemotherapy and nivolumab had higher rates of lobectomy and lower rates of pneumonectomy compared to those in the chemotherapy-alone group [14]. The NADIM II trial only recruited Stage IIIA or IIIB patients and found that nodal downstaging occurred in 72% of the patients who received neoadjuvant chemotherapy and nivolumab compared to 40% in the patients who received chemotherapy alone [45]. These findings are particularly intriguing since 66% of the patients in this study had N2 disease with 38% of them being multi-station N2 [40]. Importantly, all the patients in this trial were deemed to be surgically resectable at the time of enrollment, highlighting the role of a multidisciplinary approach in determining eligibility for one strategy or another in borderline resectable disease. Surgical downstaging is best reserved for patients who were deemed surgically resectable prior to initiation of the neoadjuvant therapy. This strategy should not be utilized to convert inoperable patients with more advanced disease into surgical candidates [59]. Of note, patient eligibility for resection and stage-specific treatments will likely change with the recent updates to the TNM classification system [60]. Although there is increasing interest in the use of the lymph node ratio for prognostication, its role in treatment decisions in early-stage surgically resectable disease has not been established [61,62,63,64].

One critical question remains: how can the field significantly increase pCR rates beyond 20–30%? The resistance to immune checkpoint inhibitors (ICIs) poses a significant challenge to many NSCLC patients. The strong and durable responses to ICIs in responders have inspired investigators to develop strategies that can overcome resistance and further harness the immune system against cancer. Applying such strategies earlier in the treatment course of surgically resectable NSCLC is likely to increase pCR rates and consequently lead to improved patient survival. Recently investigated areas included other targets such as dendritic cells and natural killer cells, and modulation of the immune microenvironment by targeting inhibitory proteins such as proprotein convertase subtilisin/kexin type 9 (PCSK9) [65,66,67,68]. In a recent analysis of TOP1501, a single-arm phase II study of perioperative pembrolizumab in 30 surgically resectable Stage IB–IIIA NSCLC patients, the mean plasma PCSK9 level was statistically significantly higher at the completion of the neoadjuvant pembrolizumab and surgery treatment. Compared to patients who showed an mPR, the PCSK9 levels were higher in those who did not show an mPR. The mean difference in baseline versus post-neoadjuvant pembrolizumab PCSK9 levels was 25.0 ng/mL (*p* = 0.0015), whereas the mean difference in PCSK9 levels following completion of neoadjuvant pembrolizumab treatment compared to the post-surgical levels was 40.2 ng/mL (*p* ≤ 0.0001) [69,70]. A phase II study evaluating the impact of neoadjuvant chemoimmunotherapy with or without a PCSK9 inhibitor is currently ongoing (NCT06385262) and will provide additional knowledge regarding PCSK9’s inhibitory immunomodulation effects. Other strategies include targeting the adenosine pathway and combination strategies including combining antibody–drug conjugates with neoadjuvant therapies. Approaches such as T cell expansion strategies, vaccine therapy, and CAR-T therapy are being investigated in advanced-stage NSCLC but have not yet been tested in early-stage NSCLC.

## 7. Conclusions

In summary, as the therapeutic landscape of surgically resectable NSCLC continues to expand, the opportunities for investigations into the best treatment approach based on patient-specific characteristics and predictors of responsiveness and/or resistance, and investigations into intensification of therapy in select patients to increase pCR rates will continue to grow. Such efforts need to be combined with multidisciplinary approaches and screening strategies that decrease the lung cancer mortality burden. The incremental strides made in the past decade, along with the promising emerging advances in medical, surgical, and radiation oncology techniques in NSCLC, have set the field up for further success.

## Figures and Tables

**Table 1:** Summary of major neoadjuvant, perioperative, and adjuvant trials incorporating checkpoint inhibition.

Study (Stage)	Study Arms	Efficacy Endpoints	mEFS/DFS/PFS (Months)	mOS (Months)	mPR (%)	pCR (%)
**NEOADJUVANT**						
CheckMate 816 Stage IB *—IIIA^+^ [17]	Nivo + CT vs. CT	1^0^: EFS and pCR 2^0^: OS	EFS: 43.8 vs. 18.4 HR: 0.66 (CI: 0.49–0.90)	mOS: NR HR for mOS: 0.71 (CI: 0.47–1.07)	36.9 vs. 8.9 OR: 5.70 (CI: 3.16–10.26)	24 vs. 2.2 OR: 13.94 (CI: 3.49–55.75)
NEOSTAR [15]	Nivo + CT vs. Ipi + Nivo + CT	1^0^: mPR 2^0^: pCR, OS, EFS	EFS: NR	NR	32.1 (80% CI: 18.7–43.1) vs. 50 (80% CI: 34.6–61.1)	18.2 (CI: 5.2–40.3) vs. 18.2 (CI: 5.2–40.3)
**PERIOPERATIVE**						
NADIM Stage IIIA ^+^ [18,19]	Single arm CT + Nivo → Nivo	1^0^: 24mo PFS 2^0^: 5 year PFS and OS	PFS: 77.1 (CI: 59.9–87.7) PFS: 65.0 (CI: 49.4–76.9)	69.3 (CI: 53.7–80.6)	83 (CI: 68–93)	63.4 (CI: 62–91)
NADIM II Stage IIIA–IIIB ^++^ [18,19]	CT + Nivo →Nivo vs. CT→ placebo	1^0^: pCR 2^0^: 24-month PFS and OS	PFS: 67.2 vs. 40.9 HR: 0.47 (CI: 0.25–0.88)	85 vs. 63.6 HR: 0.43 (CI: 0.19–0.98)	53 vs. 14 RR: 3.82 (CI: 1.49–9.79)	37 vs. 7 RR: 5.34 (CI: 1.34–21.23)
KEYNOTE 671 Stage II–IIIB ^# ++^ [20]	Pembro + CT vs. CT	1^0^: EFS and OS 2^0^: pCR and mPR	EFS: 62.4 vs. 40.6 HR: 0.58 (CI: 0.46–0.72)	64 vs. 71 HR: 0.72 (CI: 0.56–0.93)	30.2 vs. 11 Difference: 19.2 (CI: 13.9–24.7)	18.1 vs. 4.1 Difference: 14.2 (CI: 10.1–18.7)
CheckMate 77T Stage IIA–IIIB ^++^ [21]	Nivo + CT→ Nivo vs. CT→placebo	1^0^: EFS 2^0^: pCR, mPR, OS	EFS: 70.2 vs. 50 HR: 0.58 (CI: 0.42–0.81)	NR	35.4 vs. 12.1 OR: 4.01 (CI: 2.48–6.49)	25.3 vs. 4.7 OR: 6.64 (CI: 3.4–12.97)
AEGEAN (Stage II–IIIB [N2 node]) ^++^ [22]	CT + Durv →Durv vs. CT → placebo	1^0^: EFS and pCR 2^0^: mPR, DFS, OS	EFS: NR vs. 30 HR: 0.69 (CI: 0.55–0.88)	NR	33 vs. 12.3 Difference: 21 (CI: 15.1–26.9)	17.2 vs. 4.3 Difference: 13.0 (CI: 8.7–17.6)
NEOTORCH Stage II–IIIB ^++^ [23]	CT + Tori → Tori vs. CT + placebo → placebo	1^0^: IA-EFS, mPR 2^0^: IRC-EFS, OS, pCR	EFS: NR vs. 15.1 HR: 0.40 (CI: 0.28–0.57)	NE vs. 30.4 HR: 0.62 (CI: 0.33–0.76)	48.5 vs. 8.4 Difference: 40.2 (CI: 32.2–48.1)	24.8 vs. 1 Difference: 23.1 (CI: 17.6–29.8)
RATIONALE 315 [24] Stage II–IIIA ^++^	CT + TIS → TIS vs. CT + placebo → placebo	1^0^: EFS, mPR 2^0^: IRC-EFS, OS, pCR	EFS: NR for both HR: 0.56 (CI: 0.40–0.79)	NR for both HR: 0.62 (CI: 0.39–0.98)	56 vs. 15 Difference: 41 (CI: 33–95)	41 vs. 6 Difference: 35 (CI: 28–42)
**ADJUVANT**						
PEARLS/ KEYNOTE 091 [25,26] Stage IB *—IIIA ^+^	Pembro vs. placebo	1^0^: DFS in study population and PDL1 ≥ 50%	DFS ITT: 53.6 vs. 43 HR: 0.76 (CI: 0.63–0.91) DFS PDL1 ≥ 50%: NR vs. NR HR: 0.82 (CI: 0.57–1.18)	Not reported	NA	NA
IMpower 010 Stage IB *—IIIA ^+^ [27,28]	Atezo vs. BSC	1^0:^ IA-DFS in: -Stage II–IIIA (PD-L1 ≥ 1%) Stage II–IIIA PDL1 ≥ 50% -Stage II–IIIA (all PD-L1) -Stage IB–IIIA (all PD-L1)	68.5 vs. 37.3 0.7 (0.55, 0.91) NR vs. 41.1 0.48 (0.32–0.72) 57.4 vs. 40.8 0.83 (0.69–1.00) 65.6 vs. 47.8 0.85 (0.71–1.01)	NR vs. 87.1 0.77 (0.56–1.06) NR vs. 87.1 0.47 (0.28, 0.77) NR vs. NR 0.94 (0.75, 1.19) NR vs. NR 0.97 (0.78, 1.22)	NA	NA

Abbreviations: EFS, event-free survival; DFS, disease-free survival; OS, overall survival; mPR, major pathologic response, pCR, complete pathologic response; HR, hazard ratio; CI, confidence interval (all CIs were at least 95% except where indicated); OR, odds ratio; RR, relative ratio; IA-DFS: investigator-assessed disease-free survival; NR, not reached; NE, not estimable; CT, chemotherapy; Nivo, nivolumab; Ipi, ipilimumab; Pembro, pembrolizumab; Durv, durvalumab; Atezo, atezolizumab; TIS, tislelizumab; BSC, best supportive care; NA, not applicable. * Stage IB (tumors ≥ 4 cm), ^#^ with involvement of ≥1 ipsilateral mediastinal lymph node or subcarinal lymph node [N2 node stage], ^+^ AJCC 7th edition, ^++^ AJCC 8th edition.

**Table 2:** Ongoing clinical trials for immunotherapy in resectable NSCLC.

NCT Number	Study Phase	Disease Stage	Treatment Arms	Endpoints (Primary and Secondary)
NEOADJUVANT				
NCT06385262	II	Stages 1B–IIIA	A: Neoadjuvant platinum-doublet chemotherapy + cemiplimab q3 weeks ×3 cycles with alirocumab q4 weeks B: Neoadjuvant platinum-doublet chemotherapy + cemiplimab q3 weeks ×3 cycles without alirocumab	pCR ORR, DFS, OS AEs
NCT06718309	II	Stages II–IIIB	Single arm: Immunotherapy + cisplatin/carboplatin + pemetrexed OR paclitaxel ×1 cycle -> SBRT → repeat chemoimmunotherapy → resection -> maintenance immunotherapy ×1 year	pCR, mPR EFS R0 resection rate AEs
NCT04506242	II	Stages II–IIIB	Single arm: Neoadjuvant camrelizumab + apatinib ×3 cycles → resection → adjuvant camrelizumab	mPR, pCR EFS, DFS ORR AEs
NCT05800340	II	Stages IIB–IIIB	Single arm: Toripalimab + nab-paclitaxel/carboplatin OR pemetrexed/carboplatin → resection or radiation → adjuvant investigator’s choice	pCR, mPR EFS OS AEs
NCT06743581	Ib/IIa	Early Stage	Single arm: Neoadjuvant cemiplimab + dupilumab combination therapy	Safety mPR, pCR Tolerability EFS, OS
NCT04638582	II	Stages IA3–IIA	A: Neoadjuvant pembrolizumab + adjuvant pembrolizumab ± adjuvant chemotherapy B: Neoadjuvant pembrolizumab and chemotherapy + adjuvant pembrolizumab ± adjuvant chemotherapy	ctDNA resolution Radiologic response pCR rate, mPR AEs Perioperative complications
NCT05527808	II	Stages II–IIIA	Single arm: Neoadjuvant tislelizumab + pemetrexed + platinum Q3W ×2–4 cycles	mPR, pCR ORR AEs, surgery delay Minimally invasive surgery rate
ADJUVANT				
NCT06732401	III		A: Durvalumab q28d ×12 cycles B: Durvalumab and Ceralasertib q28d ×12 cycles	DFS OS AEs
NCT04267848	III		A (Active comparator): platinum doublet + observation B (Experimental): platinum doublet + sequential pembrolizumab C (Experimental): platinum doublet + combination pembrolizumab	DFS OS
NCT06528847	II	Stage IB, Grade 3	Single arm: 1200 mg benmelstobart q3w ×16 cycles	DFS OS AEs
NCT04966663	II	Early Stage	Single arm: Pemetrexed OR gemcitabine + cisplatin OR carboplatin + nivolumab	RFS
NCT06498635	III	Stages II–IIIB	A: Durvalumab—q28d ×12 cycles with CT and blood sample collection B: Active surveillance—12 mo with CT and blood sample collection	DFS EFS OS AEs
PERIOPERATIVE				
NCT06572722	II	Early Stage	A: Atezolizumab B: Nivolumab C: Pembrolizumab (random assignment in a 1:1:1 ratio)	DFS OS mPR
NCT05825625	II	Stages II, IIIA, IIIB (T3N2 only)	Single arm: SOC platinum-based chemotherapy + atezolizumab + tigoralumab ×2 cycles → surgery → SOC platinum-based chemo + atezolizumab + tiragolumab for up to 1 year	mPR, pCR Radiological response according to RECIST v1.1 EFS, OS AEs
NCT06109402	II	Stages II/IIIB (N2) NSCLC	A: Neoadjuvant immunochemotherapy + adjuvant immunotherapy B: Adjuvant immunochemotherapy + immunotherapy	ORR pCR, mPR EFS, OS 5y EFS, 5y OFS AEs, HRQol

Abbreviations: pCR, pathological complete response; mPR, major pathological response; OR, objective response; EFS, event-free survival; DFS, disease-free survival; OS, overall survival; ORR, overall response rate; RFS, relapse-free survival; AE, adverse event; ctDNA, circulating tumor DNA; HRQol, health-related quality of life. The ongoing trials were selected from the clinicaltrials.gov database. The search criteria included actively enrolling interventional trials investigating immune checkpoint inhibitors with or without chemotherapy in the neoadjuvant, adjuvant, or perioperative setting with trial sites in the United States.

## Abbreviations

The following abbreviations are used in this manuscript:

pCR	pathological complete response
mPR	major pathologic response
AE	adverse event
OR	objective response
OS	overall survival
EFS	event-free survival
DFS	disease-free survival
PFS	progression-free survival
ORR	overall response rate
RFS	relapse-free survival
WT	wild type
HRQol	health-related quality of life
NSCLC	non-small cell lung cancer
SEER	Surveillance, Epidemiology, and End Results Program
PD-L1	Programmed Death Ligand 1
TPS	Tumor Proportion Score
HR	hazard ratio
CI	confidence interval
RR	relative risk
CT	chemotherapy
TIS	tislelizumab
BSC	best supportive care
PCSK9	proprotein convertase subtilisin/kexin type 9
